# Incorporation of shear wave elastography into a prediction model in the assessment of cervical lymph nodes

**DOI:** 10.1371/journal.pone.0221062

**Published:** 2019-08-15

**Authors:** Wu-Chia Lo, Wan-Lun Hsu, Chi-Te Wang, Po-Wen Cheng, Li-Jen Liao

**Affiliations:** 1 Department of Otolaryngology, Far Eastern Memorial Hospital, Taipei, Taiwan; 2 Genomics Research Center, Academia Sinica, Taipei, Taiwan; 3 Department of Electrical Engineering, Yuan Ze University, Taoyuan, Taiwan; Wayne State University, UNITED STATES

## Abstract

**Rationale and objectives:**

To assess the performance of shear wave elastography (SWE) and an extended model in predicting malignant cervical lymph nodes (LNs).

**Materials and methods:**

109 patients who underwent ultrasound (US) and SWE before needle biopsy were enrolled. The optimal cutoff value of elasticity indices (EIs) was determined by receiver operating characteristic (ROC) curves. The c-statistic, net reclassification improvement (NRI) and integrated discrimination improvement (IDI) were used to compare extended model and traditional one.

**Results:**

Malignant LNs had higher EIs than benign nodes (p < 0.001). The optimal cutoff point was 42 kilopascal, corresponding to 83.3% sensitivity, 64.7% specificity, and 68.8% overall accuracy. A multivariable logistic regression analysis confirmed that EI was an independent predictor for malignancy. The new extended prediction model had a positive NRI (0.96) and IDI (0.10) for predicting malignant neck LNs. Nevertheless, the c-statistic was not significantly different between the two models.

**Conclusion:**

The parameter of SWE theoretically improve the model performance. However, its real clinical impact is minor, as the parameters of US-based model is already very robust. SWE can be considered as an adjunctive quantitative tool beyond conventional US examination.

## Introduction

Lumps found in the neck can be a signal of potential malignancy, and the presence of malignant neck lymph nodes (LNs) can lead to a poor prognosis. Therefore, it is important to distinguish malignant from benign LNs as soon as possible. Ultrasound (US) is usually used as the first tool to evaluate cervical LNs. Sonographically, malignant LNs tend to be larger and rounder, have ill-defined contour and bizarre echo-texture, and show loss of echogenic hilus and atypical vascular patterns [1, 2]. However, no individual US characteristic is specific to malignancy. Previously, we published a real-time predictive scoring model for predicting malignant LNs and showed greater than 80% overall accuracy [3]. The formula of the prediction model was “0.06 x age + 4.76 x Short-axis (S)/Long-axis (L) ratio + 2.15 x internal echo (homogenous score 0; heterogeneous score 1) + 1.80 x vascular pattern (avascular or hilar type score 0; others score 1)”. Neck LNs were regarded as positive when they scored ≧7. This prediction model was further validated to have good probability results at another institution [4].

Recently, elastography has been emerged as a useful complementary tool during ultrasound scanning. Several methods and scores are currently utilized to evaluate the stiffness of LNs, such as real-time elastography (RTE), strain elastography, and shear wave elastography (SWE). Other modalities such as magnetic resonance elastography are also used for elasticity imaging [5]. Elastograms are images of tissue stiffness and may be in color, grayscale, or a combination of the two [5, 6]. SWE is a quantitative technique and it may increase the diagnostic confidence of less experienced operators during performing head and neck US [7].

Lately, several studies have revealed the utility of quantitative SWE in the workup of neck LNs [8–14]. In 2012, Bhatia et al. reported an optimal cutoff value of 30.2 kilopascal (kPa) of SWE was feasible to evaluate cervical LNs, corresponding to sensitivity, specificity, and accuracy of 42%, 100%, and 62%, respectively [10]. Latterly, Desmots et al. (2016) demonstrated that the combination of SWE and B-mode US had the tendency to increase diagnostic accuracy [14]. However, SWE, as a new technique, is needed to be assessed to have additional value over conventional US [15]. The aim of this study was to assess the usefulness of SWE combined with conventional US predictors, compared with traditional predictive model, in the prediction of malignant cervical LNs.

## Materials and methods

### Patients

**Fig 1:**
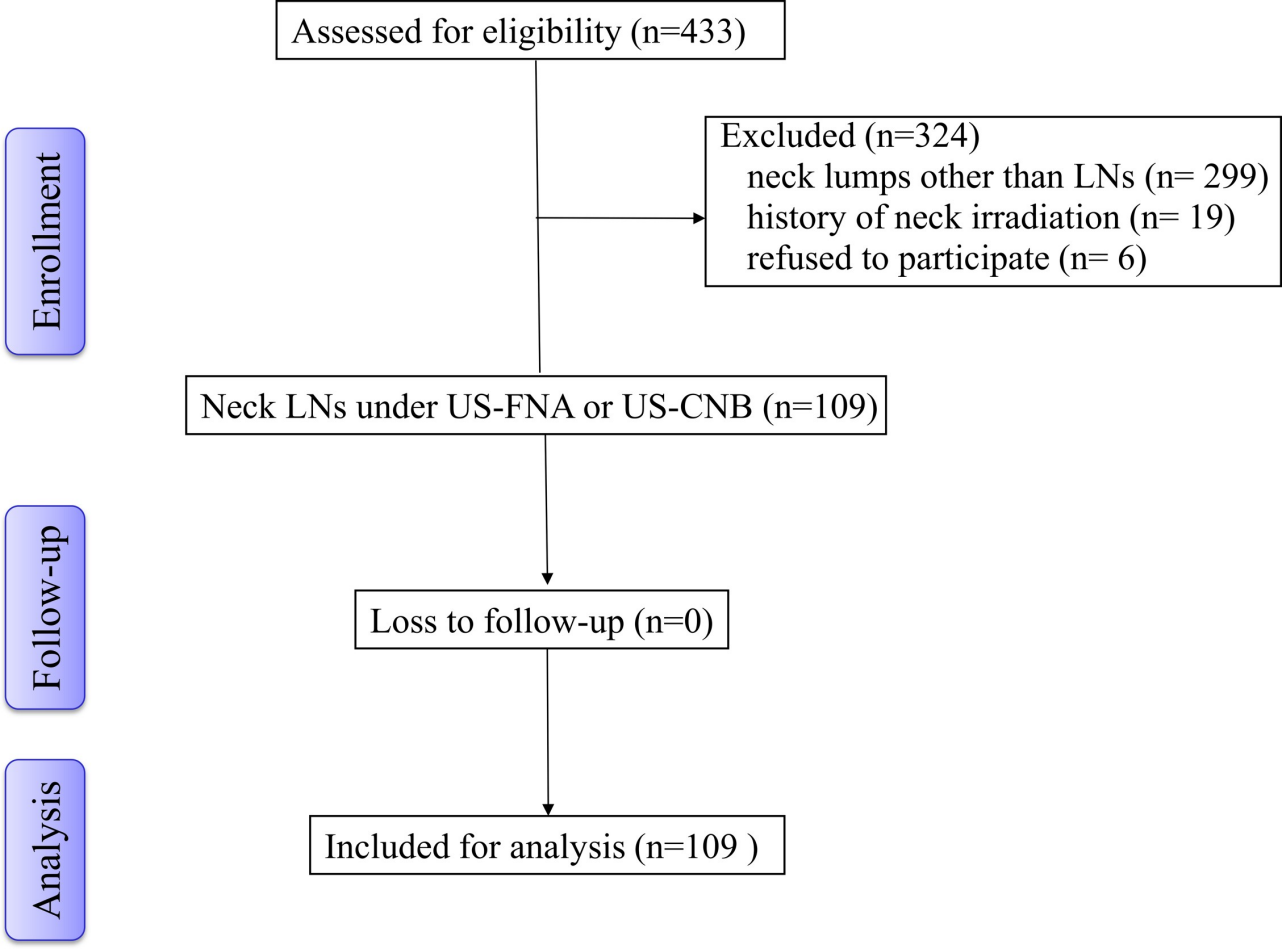
The flow chart of the selection algorithm of the subjects.

This study was first approved by the institutional ethics review board of local ethics committee (Far Eastern Memorial Hospital- Institutional Review Board -105021-E). We prospectively recruited patients who underwent gray-scale US, power Doppler US and SWE prior to US-guided fine-needle aspiration (US-FNA) or core needle biopsy (US-CNB) from Aug 2015 to Jan 2016. These patients were scheduled for US exam due to the presentation of palpable neck masses. Each patient had written informed consent signed before participation in the study. The patient exclusion criteria were as follows: neck lumps other than LNs and a history of irradiation in the head and neck region. To end, a total of 109 adult patients were included in this study (Fig 1). Of them, 31 patients had history of cancers, including newly-diagnosed cancer patients in 14 and treated patients in 17. In treated patients, all had treatment for the primary site and 7 patients had received neck dissection.

### Conventional ultrasonography

The Toshiba Aplio 500 US system (Otawara, Japan) with a 5–14 MHz linear probe was used to examine the neck. Two experienced head and neck surgeons and sonographers (LJ Liao & WC Lo, who both had more than 7 years of experience in US) performed the exams. At first, the targeted LN was assessed with gray-scale sonography for morphologic parameters, both in horizontal and longitudinal sections. The short-axis (S) and long-axis (L) diameters, and S/L ratio were measured. Echogenicity with respect to the surrounding soft tissue was assessed and classified as hypoechoic, isoechoic, or hyperechoic. We defined nodal margins as regular or irregular. Echogenic hilus was classified as its presence or absence. Internal echo pattern was divided into heterogeneous or homogeneous. The power Doppler US was set for high sensitivity with a low wall filter to allow for the detection of vessels with low blood flow. Color gain standardization was optimized according to Bude and Rubin’s description [16]. Vascular patterns were categorized as avascular or hilar type versus mixed, spotted, or peripheral type.

### Shear wave elastography (SWE)

**Fig 2:**
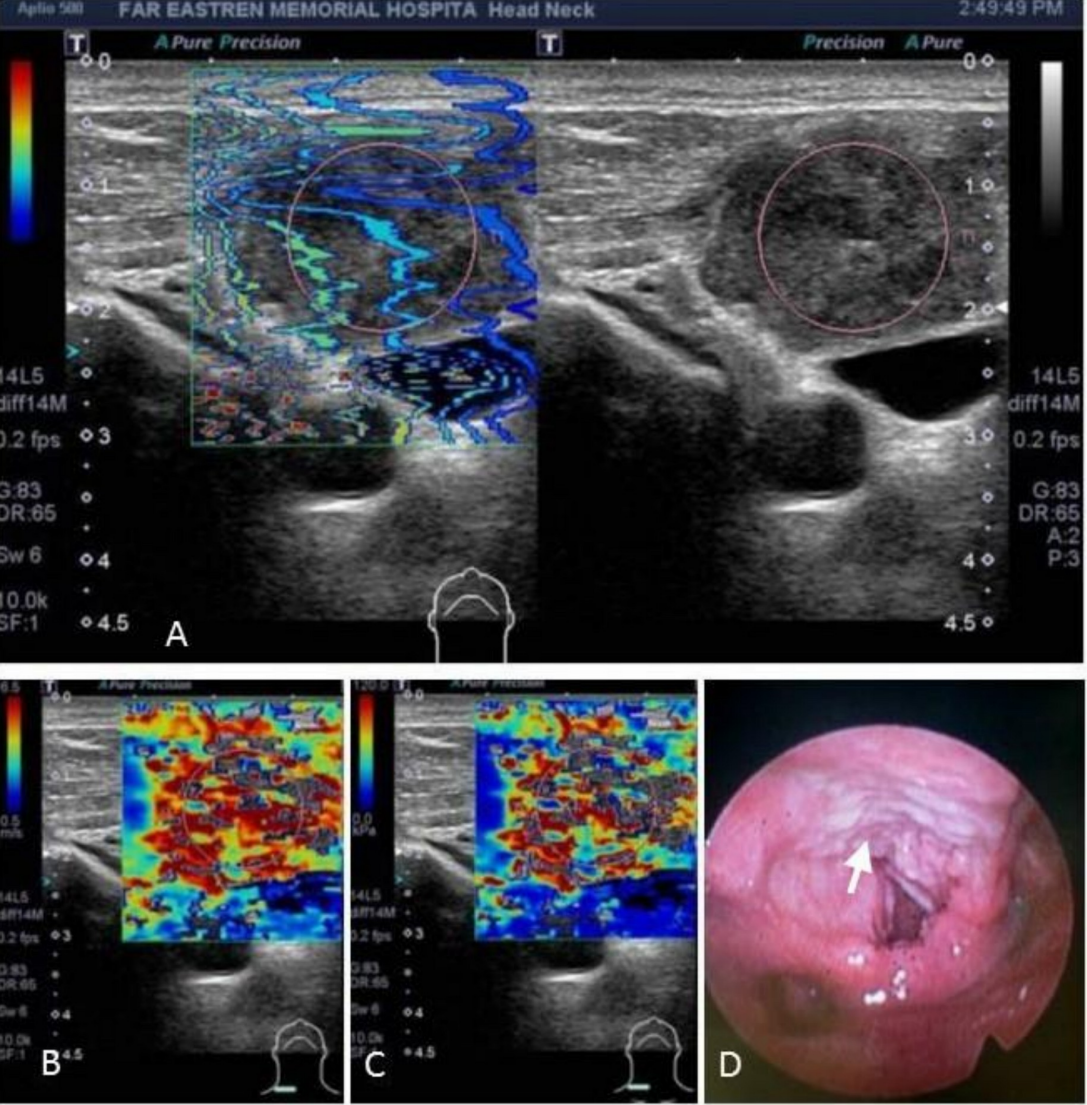
A fifty-three year-old man presented with palpable LNs at right neck. SWE was used to evaluate the stiffness of the targeted LN (A: Propagation mode, B: Speed mode: 5 m/s; C: Young’s modulus: 76.1 kPa). US-FNA was performed and the cytology report was positive for carcinoma. He was finally proved to have a laryngeal cancer (D, arrow).

In performing SWE, the node that beneath the probe was deformed by a "push pulse" generated from the probe. Then the velocity of the shear waves propagating within the tissue was detected, and the stiffness was assessed based on the detected shear velocity. It was possible to observe whether the shear waves propagated properly in a single still image displayed in propagation (arrival time contour) mode (**Fig 2A**). In areas where the contour lines were parallel, the shear waves propagated properly ensuring the reliability of the obtained data. Then, we could shift to speed mode (shear velocity, m/s) and Young’s modulus (**Fig 2B & 2C**). In Young’s modulus, the stiffest region within the node was selected by visual inspection according to the color-coded elastogram and elasticity indices (EIs, kPa) were measured within an approximately 5–10 mm circular region. EIs used in this model corresponds to the maximum Young's modulus of the region of interest (ROI). Regions of interest were set in the stiffest areas to obtain the maximum Young’s modulus was according to the protocol reported by Choi et al. [11]. The reason was based on the hypothesis that it was better than the mean Young’s modulus in assessing LNs with focal cortical metastases or with focal necrotic areas within the nodes.

### US-guided needle procedures

The largest or the most doubtful LN was chosen for the needle procedure. US-guided procedure was carried out with the same array probe guiding the placement of a 22-gauge fine needle or 18-gauge core needle within the node. Six passes in the lesion was made during FNA or 2–3 pieces of tissues were took after CNB to obtain sufficient material for assessment. The final malignant diagnoses were made by core-needle biopsies or US-FNA studies combined with a biopsy-proven primary site malignancy (**Fig 2D**). The patients with negative cytological results were followed for at least 6 months to confirm that no malignancy had developed among these nodes.

### Statistical analysis

Student t and Chi-squared tests were used to determine the differences in clinical parameters (i.e. age, sex, side and level of occurrence, diameter of short and long axes, S/L ratios, internal echo, echogenicity, margin, echogenic hilus, vascular pattern and EIs) between benign and malignant LNs. A p-value < 0.004 was interpreted as statistically significant after adjusting the α-error by Bonferroni test. The optimal cutoff point of EIs was determined at the point of highest accuracy for predicting malignancy by receiver operating characteristic (ROC) curve analyses [17]. The diagnostic performance was expressed as sensitivity, specificity, positive predictive value (PPV), negative predictive value (NPV), overall accuracy, and the area under the ROC curve (AUC, C-statistic). The extended prediction model (traditional model + SWE) was created based on a logistic regression analysis [3]. C-statistic analysis was used for comparison between the extended and traditional models and a p-value < 0.05 was interpreted as statistically significant. For the measurement of improvement in risk prediction, net reclassification improvement (NRI) and integrated discrimination improvement (IDI) were used to compare the new extended prediction model with the traditional one [18, 19]. If the 95% confidence interval (CI) of the NRI and IDI did not include zero, then the results showed significant improvement in risk prediction. All statistical analyses were accomplished using Stata software, version 12.0 (Stata Corp. LP, College Station, TX)

## Results

A total of 109 adults, including 55 females (50.5%) and 54 males (49.5%), with 109 cervical LNs were recruited in the study. The mean age was 46 years (range, 21–86 years). Among them, 24 LNs (22%) were diagnosed to be malignant and 85 LNs (78%) were found to be benign (see S1 Table). In patients who had malignant LNs, there were 6 oral cancers, 3 nasopharyngeal carcinomas, 2 oropharyngeal cancers, 2 hypopharyngeal cancers, 2 carcinomas of unknown primary, 2 lymphomas, 2 endometrial cancers, 1 melanoma and 1 laryngeal, esophageal, lung, and prostate cancer.

**Fig 3:**
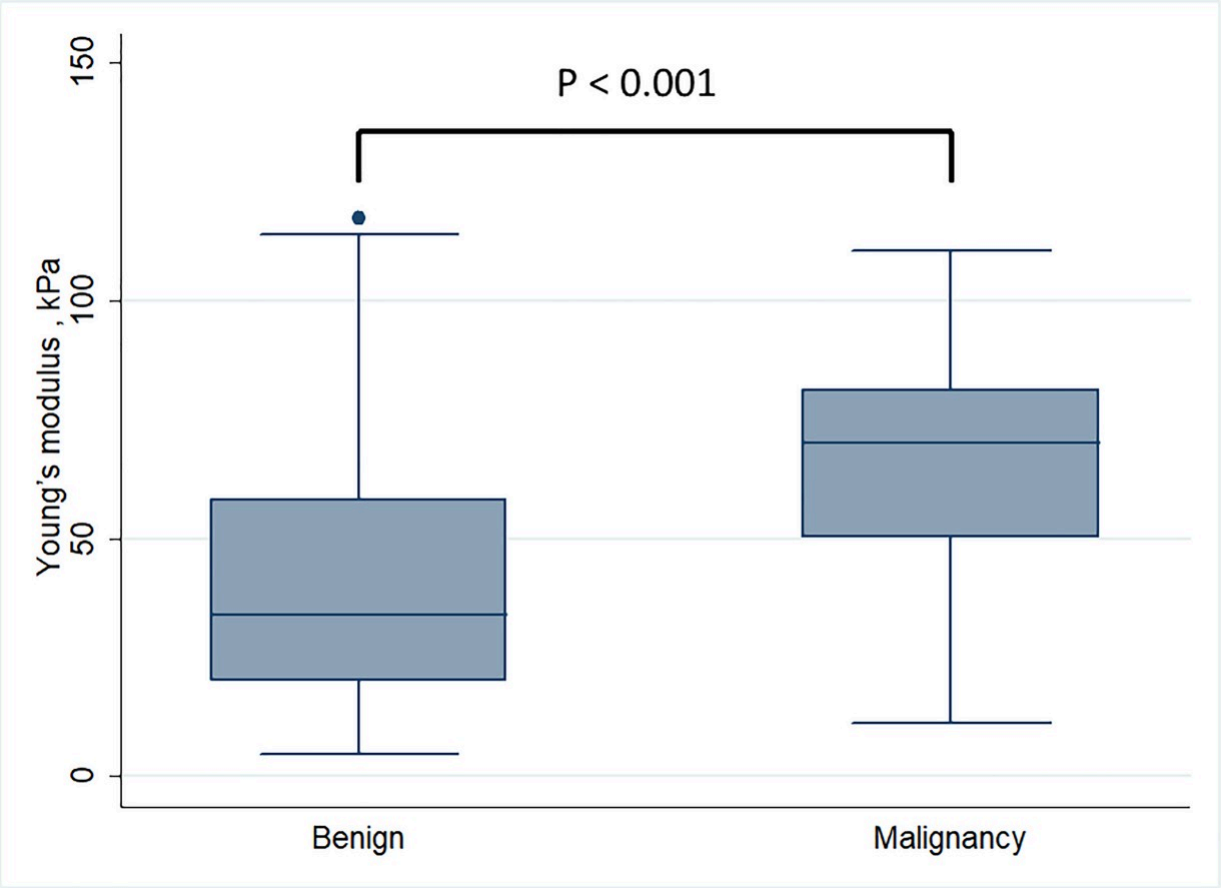
Comparison of Young’s modulus values between benign and malignant nodes (41.4 ±26.5 versus 66.3±24.3 kPa, p<0.001).

Comparisons of demographic and US parameters between malignant and benign LNs were shown in **Table 1**. Age, gender, short and long axis, S/L ratio, boundary, internal echo, echogenic hilus, vascular pattern and EIs were significantly different between malignant and benign nodal disease (*p* < 0.004). Malignant LNs had higher Young’s modulus values (66.3 ± 24.3 kPa) than benign LNs (41.4 ± 26.5 kPa) (p < 0.001, **Fig 3**).

**Table 1:** Demographic and sonographic findings between malignant and benign LNs.

	Benign (n = 85)	Malignant (n = 24)	p-value
Age	43.2±13.9	54.0±11.1	<0.001^‡^
Gender (Female/Male)	51/34	4/20	<0.001^#^
Side (Left/Right)	37/48	12/12	0.574^#^
Level (1&5/2-4)	63/22	8/16	<0.001^#^
Size-short axis (cm)	0.66±0.27	1.64±0.79	<0.001^‡^
Size-long axis (cm)	1.32±0.53	2.47±1.08	<0.001^‡^
Shape-S/L ratio	0.53±0.21	0.69±0.18	0.001^‡^
Boundary (Regular/Irregular)	81/4	11/13	<0.001^#^
Internal echo (Homo-/Hetero-geneous)	81/4	7/17	<0.001^#^
Hilar echo (Absent/Present)	38/47	22/2	<0.001^#^
Vascular pattern (avascular&hilar/other)	75/10	12/12	<0.001^#^
EIs (kPa)	41.4±26.5	66.3±24.3	<0.001^‡^

EIs: elasticity indices; S: short-axis; L: long-axis; kPa: kilopascal

^‡^:Student’s t test

^#^:Chi-squared test

**Fig 4:**
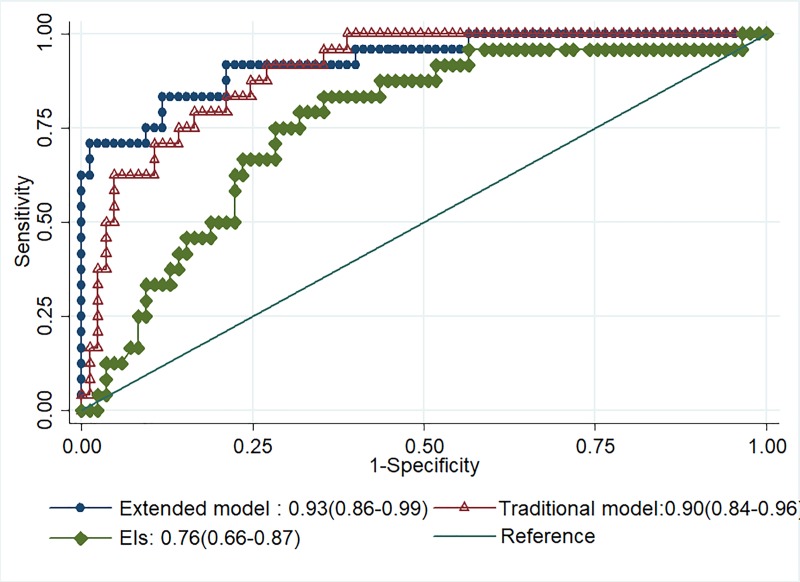
Comparison of the diagnostic performances in predicting malignant LNs among the three models: Elasticity indices, the traditional model, and the extended model containing SWE. The AUC of the traditional model (0.90, 95% CI: 0.84–0.96) and extended model (0.93, 95% CI: 0.86–0.99) were superior to EIs (0.76, 95% CI: 0.66–0.87) (ps < 0.02). The c-statistic between the traditional model and extended model was not statically different (p = 0.26).

The prediction models were compared using ROC curve analyses. An EI ≧ 42 kPa was the best cutoff point for prediction of malignancy, corresponding to sensitivity of 83.3%, specificity of 64.7%, PPV of 40%, NPV of 93.2%, and overall accuracy of 68.8% (**Table 2 & Fig 4**). Our previously proposed (traditional) model had a diagnostic performance of 79.2% sensitivity, 82.4% specificity, 55.9% PPV, 93.3% NPV, and 81.7% overall accuracy in predicting malignant LNs. A multivariable logistic regression analysis confirmed that an EI ≧42 kPa was an independent predictor for malignancy (OR 9.86, 95% CI: 1.82–53.4, p < 0.01). According to the results of the logistic regression, an extended prediction model (traditional model + SWE) using the parameters and their regression coefficients in combination was proposed as: 0.04 x (age) + 2.28 x (S/L ratio) + 3.42 x (internal echo) + 2.29 x (EIs). The best cutoff point to differentiate benign from malignant LNs according to ROC analysis was 5.80, corresponding to sensitivity of 83.3%, specificity of 88.2%, PPV of 66.7%, NPV of 94.9%, and overall accuracy of 87.2%.

**Table 2:** Comparisons of diagnostic performances of three models for diagnosing malignant lymph nodes.

Parameters (cutoff)	Sensitivity (%) [95% CI]	Specificity (%) [95% CI]	PPV (%) [95% CI]	NPV (%) [95% CI]	Accuracy (%) [95% CI]
EIs	83.3 [68.4–98.2%]	64.7 [54.5–74.9%]	40.0 [26.4–53.6%]	93.2 [86.8–99.6%]	68.8 [60.1–77.5%]
Traditional model ^ψ^	79.2 [62.9–95.4%)	82.4 [74.2–90.5%]	55.9 [39.2–72.6%)	93.3 [87.7–99.0%]	81.7 [74.4–88.9%]
Extended model ^ζ^	83.3 [68.4–98.2%]	88.2 [81.4–95.1%]	66.7 [49.8–83.5%]	94.9 [90.1–99.8%]	87.2 [80.9–93.4%]

EIs: elasticity indices

^ψ^ Traditional model = 0.06 x (age) + 4.76 x (S/L ratio) + 2.15 x (internal echo) + 1.8 x (vascular pattern)

^ζ^ Extended model = 0.04 x (age) + 2.28 x (S/L ratio) + 3.42 x (internal echo) + 2.29 x (EIs)

Compared to the traditional model, the extended model by adding SWE as a new predictor resulted in a category free NRI of 0.96 (95% CI: 0.54–1.33) and an IDI of 0.10 (95% CI: 0.03–0.26), indicating positive improvement. However, C-statistic analysis between the extended and traditional models revealed no significant difference (p = 0.26).

## Discussion

The sonographical parameters for differentiating malignant from benign cervical LNs have been well documented, including size, shape, echogenic hilus, internal echo, and margin in B-mode sonography, as well as vascular pattern in Doppler US [1, 2, 20]. It is well known that LN size positively correlates with a greater chance of malignancy. In this study, when a malignant LN was diagnosed, the mean short- and long-axes diameter was 1.64 and 2.47 cm, respectively. Under US, shape is usually described by determining the LN’s S/L ratio. It is also evident that the S/L ratio of a LN greater than 0.6 indicates a round shape, which is more likely to be malignant. In comparison, a LN with the S/L ratio below 0.6 designates an oval shape and tends to be benign. Additionally, a benign LN usually has an echogenic hilus, homogeneous cortex and smooth margin, whereas a malignant LN is apt to lack echogenic hilum and has heterogeneous appearance with an irregular border. Furthermore, Doppler imaging is an another US tool that used to discriminate malignant LNs from benign ones. Most benign LNs have avascular or hilar type vascular patterns, while malignant LNs are probable to possess spotted, peripheral or mixed type vascular patterns [2].

In the past, we had developed a prediction model based on a patient’s age, grey-scale features, and Doppler image patterns to predict a malignant LN. Subsequently, internal and external validations were performed to test this predictive scoring model and promising results were reported [3, 4]. The formula was programmed into a synchronized, computerized sonographic reporting system in our hospital. Recently, elastographic images generated by real-time supersonic shear wave and quantitative measurements within static elasticity image frames could be performed. In the present study, there was a significant difference in EIs between malignant and benign nodes. In multivariable logistic regression, EIs was still an independent predictor for malignant nodes. In addition, ROC analysis showed that cutoff of 42 kPa had diagnostic values of 83.3% sensitivity, 64.7% specificity, and 68.8% overall accuracy. When SWE was combined into the abovementioned traditional prediction model, it resulted in a category free NRI of 0.96 (95% CI: 0.54–1.33) and an IDI of 0.10 (95% CI: 0.03–0.26). As a result, we found that SWE was an independent predictor of malignant LNs and could positively improve the predictive model.

**Fig 5:**
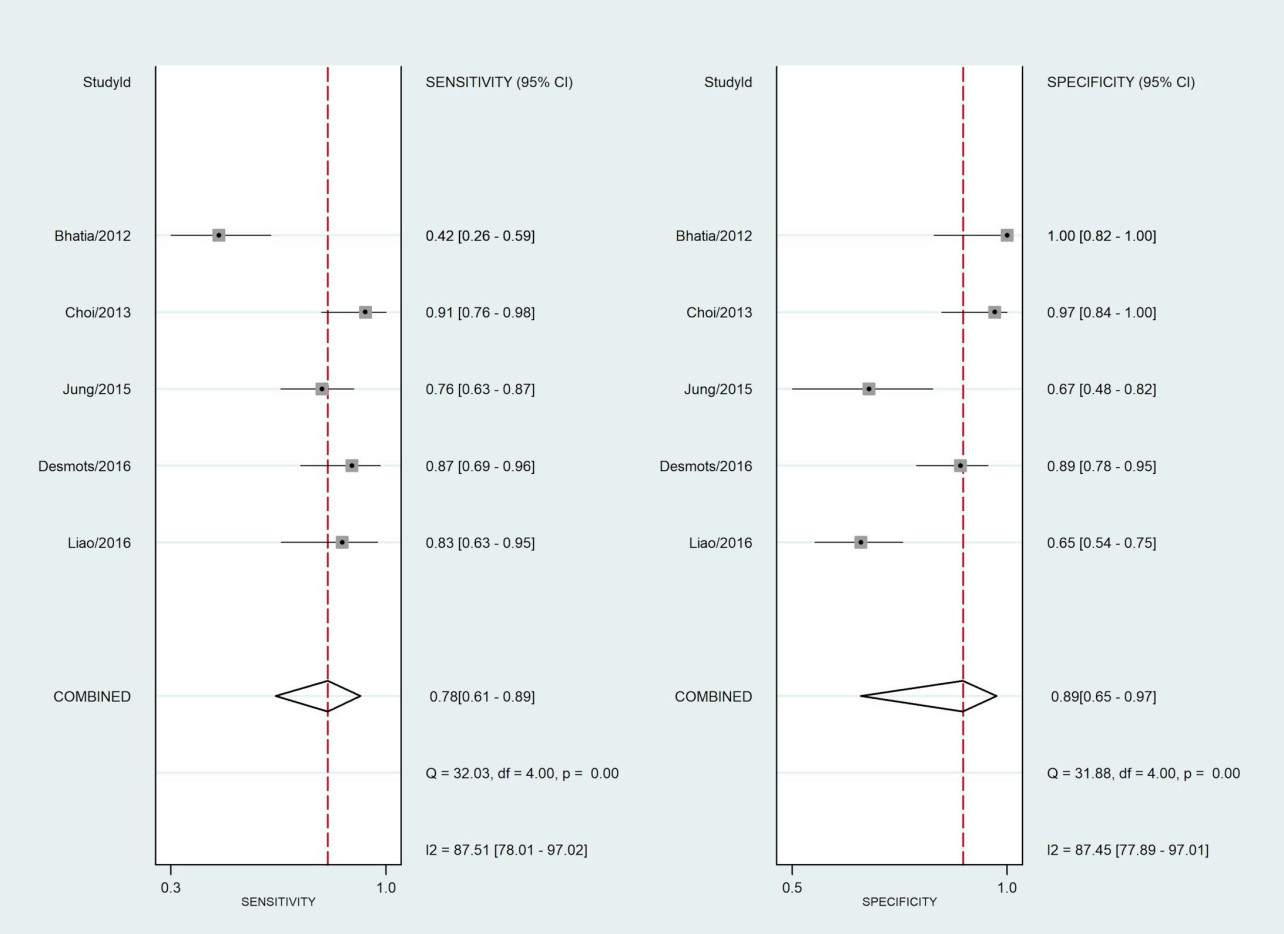
A summary of the diagnostic performances of SWE of cervical LNs in the current and published studies. Using a bivariate model, the pooled sensitivity was 78% (95% CI: 61–89%) and the pooled specificity was 89% (95% CI: 65–97%).

To further examine the utility of SWE, four other reported studies (see S2 Table) using SWE to examine a LN were summarized in **Table 3**and **Fig 5**[10, 11, 14, 21]. We used a bivariate model to pool the diagnostic performances [22]. In diagnosing malignant neck LNs, the pooled sensitivity of SWE was 78% (95% CI: 61–89%) and the pooled specificity was 89% (95% CI: 65–97%). Significant heterogeneity (I-squared = 88%, 95% CI: 78–97%) was found, and different cutoff points were reported among these studies. These variations may be due to different patient populations, different US machines, and different elasticity parameters chosen by operators. Some chose to report the mean Young's modulus, which strongly depends on the ROI size, especially in tissues with different structures like cervical area. The ROI size are not equal and depends on particular patient.

**Table 3:** Summary of published studies on SWE for evaluation of neck LNs and the pooled diagnostic performance by using a bivariate random effect model.

No.	Author	Year	n	Patients	Nodes	Benign	Malignant	Cutoff	Sensitivity	Specificity
1	Bhatia[10]	2012	46	Mixed	55	24	31	30kPa	42%	100%
2	Choi[11]	2013	15	HNC	67	33	34	19kPa	91%	97%
3	Jung[21]	2015	66	PTC	84	33	51	29kPa	77%	67%
4	Desmots[14]	2016	56	Mixed	92	62	30	31kPa	87%	88%
5	Current study		109	Mixed	109	85	24	42kPa	83%	65%
Pooled Summary			294		407	237	170		78% (95% CI: 61–89%)	89% (95% CI: 65–97%)

HNC: head and neck cancer; PTC: papillary thyroid carcinoma

In this study, the traditional prediction model was validated again with a diagnostic performance of 79.2% sensitivity, 82.4% specificity, and 81.7% accuracy in predicting malignant LNs. In comparison, the new extended one showed positive improvement in prediction by using the NRI and IDI, although the c-statistic was not significantly different between the two models. However, it should be noted that c-statistic is not sensitive enough to detect small improvements in model performance when a new marker is added to a model that already includes important predictors [18]. To overcome this problem, we used the continuous NRI and IDI index as the measures of discrimination between the two models. The NRI quantifies the numbers of individuals that are correctly reclassified into clinically meaningful higher or lower risk categories with the addition of a new predictor, while the IDI summarizes overall possible risk thresholds by estimating improvement in the average sensitivity minus the decrease in the mean specificity [18, 19]. To the best of our knowledge, there is only one study has incorporated SWE into the predictive scoring system and showed that SWE does not add much new predictive power than the combination of epidemiologic and classic ultrasound parameters [23].

This study had several limitations. Firstly, the numbers of patients included in this study were still limited. Secondly, we did not compare real-time elastography and SWE at the same time. Third, we did not focus on one histological type or primary site and HPV status was not evaluated in this study, these factors might have influences on the predictor of age in the prediction model. Fourth, the final diagnoses were not all made from the results of histopathology. After the follow-up of at least 6 months, patients who had reduced or equal nodal size were considered benign. Unavoidably there may be minute chance that the node was positive when slow-growing metastatic lesion, such as metastasis from papillary thyroid carcinoma, was encountered. Similarly, most malignant nodal diagnoses were based on definitive histology (n = 19) but small portion of final malignant nodal diagnoses (n = 5) were built on FNA studies combined with a biopsy-proven primary site malignancy. However, the diagnoses of malignant nodes in these patients were also referred to other image studies such as MRI or CT and the treatment response. Finally, the issue of reproducibility is not addressed in this study.

## Conclusions

In conclusion, the parameter of SWE theoretically improve the model performance. However, its real clinical impact is minor, as the parameters of US-based model is already very robust. SWE can be considered as an adjunctive quantitative tool beyond conventional US examination.

## Supporting information

S1 Table SWE LN raw database. (XLS)

S2 Table Meta raw database. (XLSX)
